# Sagittal balance in the conservative treatment of pathological kyphosis: a retrospective observational study

**DOI:** 10.1186/1748-7161-8-S2-O29

**Published:** 2013-09-18

**Authors:** Salvatore Minnella, Sabrina Donzelli, Monia Lusini, Fabio Zaina, Michele Romano, Alessandra Negrini, Stefano Negrini

**Affiliations:** 1 ISICO Italian Scientific Spine Institute, Milan, Italy

## Background

Sagittal alignment of the spine is not well understood. Some authors have demonstrated that the spinal shape is closely correlated with the pelvic shape and orientation. Uncertainty exists if pathological kyphosis should be treated, regardless of sagittal balance.

## Purpose

The goal of this study was to assess how conservative treatment of pathological kyphosis during growth correlates with spinal and pelvic sagittal balance parameters.

## Methods

Study Design: Retrospective observational study. Population: A total of 23 patients (15 males; 8 females), aged 11 to 17 years were included. Each patient had at least two clinical evaluations and spinal X rays (lateral projection) at the time of therapy start (T0) and stop (T1). Methods: 20 patients presented thoracic hyperkyphosis and three presented thoracolumbar kyphosis. All of the patients were treated conservatively; specifically, 17 were treated with brace plus specific exercises and 6 were treated with only specific exercises. For all of the patients we measured the sagittal parameters of thoracic kyphosis (TK), lumbar lordosis (LL), pelvic incidence (PI), pelvic tilt (PT), sacral slope (SS) and spinosacral angle (SSA). Statistical analysis: paired Wilcoxon test and correlation (Pearson).

## Results

Mean and standard deviation for each parameter were measured in T0: TK, LL, PI 43.83± 7.95, PT 4.83± 6.96, SS 38.91± 7.67, SSA; and in T1: TK, LL, PI 41.87± 9.62, PT 5.48± 7.04, SS 36.39± 7.80, SSA. Highly significant improvements were found for the main spinal parameters from T0 to T1: TK reduced from 54.22± 13.58 to 44.48± 12.80 (p= 0.0006), LL from 57.43± 11.16 to 51.13± 10.28 (p= 0.0077) and SSA from 132.87± 9.80 to 127.09± 9.24 (p= 0.0036); no significant differences were found for the other parameters. Looking at sagittal parameter correlations,very few were found at the start of treatment: SS correlated with LL (R=0.651; p=0,007), SSA (R=0.600; p=0.0025) and PI (R=0.596; p=0.0027) which also correlated with PT (R=0.515; p=0.0119). Contrarily, at the end of treatment, most parameters were correlated: LL with PI ( R=0.514; p=0.0122), SS (R=0.499; p=0.0152), and SSA (R=0.743; p<0.0001), PI with PT(R=0.600; p=0.0024), SS (R= 0.691; p=0.0003) and SSA (R=0.628; p=0.0013) and SS with SSA (R=0.650; p=0.0008).

## Conclusions and discussion

According to our results, conservative treatment is an effective therapy for pathological kyphosis. Since it can change correlations between sagittal balance parameters, making them similar to those described in previous studies in healthy subjects, we can hypothesize its primary role in restoring sagittal balance itself.
